# UHV-based analytics with electrochemical oxygen activity control

**DOI:** 10.1039/d5ta02648b

**Published:** 2025-07-31

**Authors:** Andreas Nenning, Stanislaus Breitwieser, Christian Melcher, Jürgen Fleig

**Affiliations:** a Institute of Chemical Technologies and Analytics, Research Group for Electrochemical Energy Conversion TU Wien Austria andreas.nenning@tuwien.ac.at

## Abstract

The (electro)chemical properties of electrode materials in solid oxide cells or oxide-based redox catalysts are determined by the surface chemistry of these materials under operation conditions. Surface point defect concentrations strongly depend on the oxygen stoichiometry in the bulk and the gas phase's chemical composition (*e.g.*, oxygen activity). However, many chemically sensitive surface analysis techniques rely on UHV conditions, leading to a two-fold deviation from surfaces under operational conditions. On the one hand, adsorbed gas phase species are missing in UHV. On the other hand, transition metal oxidation states and the oxygen vacancy concentration at surfaces are connected to the oxygen stoichiometry in the bulk of the material, which is inevitably altered during cell transfer from electrochemical measurement to UHV-based analytics. To reduce this two-fold gap between analytical studies and typical operation conditions, we present a novel solid oxide cell design for electrochemical oxygen activity control of surfaces in UHV-based analytic tools. Its key feature is an oxygen-ion buffering counter electrode containing a Fe|FeO phase equilibrium with known oxygen activity. A defined voltage between this counter electrode and the oxide under investigation (used as working electrode) defines the oxygen activity of the relevant oxide surface. Moreover, simultaneous thin film coulometry allows the determination of the bulk oxygen deficiency in the respective oxides. As a proof of concept, we use UHV-based XPS to compare the bulk and surface reducibility of fluorite-type Gd-doped ceria and perovskite-type Fe-doped SrTiO_3_ under electrochemical oxygen activity control. We show that the cell voltage can tune the transition metal oxidation states and oxygen vacancy concentration at the surface. These relate well to the actual solid oxide cell operation at the same temperature and *p*(O_2_).

## Abstract 2

With the right electrochemical cell design, the oxygen activity in an oxide can be controlled precisely, even for UHV-based surface analytics.

## Graphical abstract

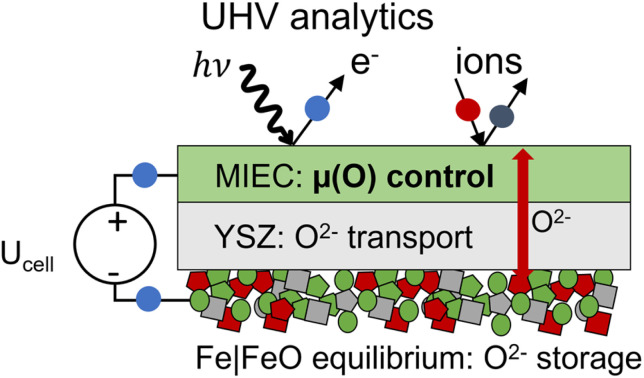

## 1. Introduction

Catalytically active and electrically conductive oxides with mixed transition metal oxidation states and significant oxygen nonstoichiometry are used as electrodes in solid oxide fuel and electrolysis cells,^1–3^ and they are also rather common for heterogeneous catalysis of redox reactions, especially when they rely on the Mars-Van-Krevelen mechanism.^4,5^ The bulk and surface chemistry of these materials depend strongly on the experimental conditions (temperature, oxygen partial pressure, but also prehistory, *e.g.* previous annealing steps). All of these have a significant impact on the electrochemical or catalytic activity of the surfaces. Accordingly, investigating the composition and oxidation states of oxide surfaces under operation conditions is essential for better understanding the corresponding (electro-)catalytic properties.

**Fig. 1:**
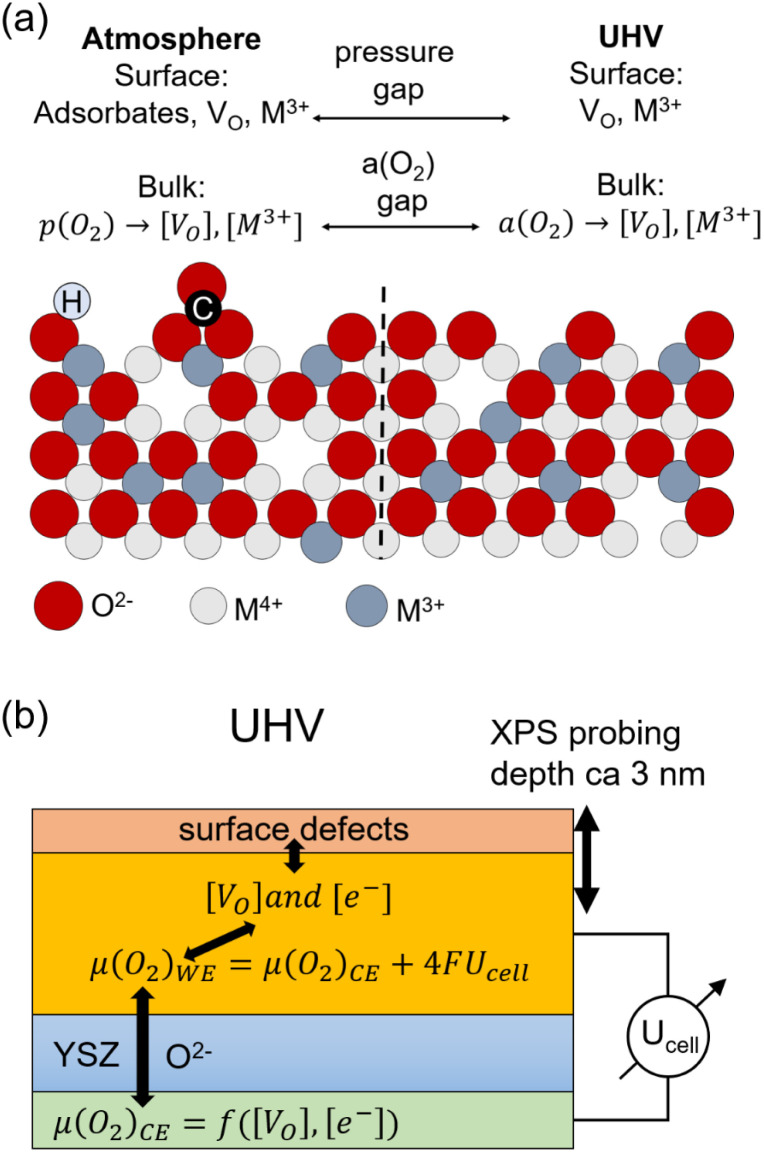
(a) Simplified sketch of surface and bulk defects at an oxygen MO_2−*δ*_ surface sample in (left) atmosphere and (right) vacuum. (b) Schematic of the coupling of the oxygen chemical potentials and point defects in a solid oxide cell in UHV.

From a viewpoint of heterogeneous catalysis, one might think that the surface chemistry of these oxides is solely determined by gas phase and temperature, which determine the concentrations of adsorbed species and reaction intermediates. However, the defect chemical part of the surface chemistry exhibits a more complex dependence. At elevated temperatures, the concentrations of point defects, especially oxygen vacancies [V_O_] and reduced metal ions ([M^3+^] in a MO_2_ oxide) in the surface and bulk are usually in thermodynamic equilibrium.^6^ This does not necessarily mean that they have equal concentration.^11^ If gas phase and oxide are in thermodynamic equilibrium, the gas phase *p*(O_2_) determines the bulk defect chemistry, represented by concentrations of reduced transition metal ions and oxygen vacancies ([V_O_] and [M^3+^]). However, these defect concentrations are not directly connected to surface adsorbates, but more generally linked to the oxygen activity or chemical potential of the gas phase. This is sketched for an oxygen-deficient MO_2−*δ*_ surface in Fig. 1a.

If that oxide is an electrode in an electrochemical cell, application of a voltage will lead to an oxygen ion current across the electrode/electrolyte interface. This oxygen ion current modifies the V_O_ and M^3+^ concentrations, and thus the oxygen chemical potential or oxygen activity in the electrode material, thereby breaking the equilibrium between gas phase and oxide. The electrochemically altered concentrations V_O_ and M^3+^ concentrations in the bulk and also an effect on the surface chemistry as exemplified in many ambient pressure XPS studies.^6–10^ In summary, two factors influence the surface chemistry: the gas phase and the bulk oxygen chemical potential. Gas phase molecules may adsorb and form, *e.g.* hydroxyl, carbonate, or O_2_^*x*−^ species. The oxygen chemical potential in the oxide electrode, however, is a function of gas phase and cell voltage.

Unfortunately, many sensitive surface analysis tools operate only in UHV. Typically, catalysts are being cooled down to room temperature and investigated *ex situ* by UHV-based surface analytics. This causes a two-fold gap between UHV measurement conditions and typical operation conditions, sketched in Fig. 1a: absence of reaction intermediates due to a gap in the gas phase pressure (often discussed in literature as the “pressure gap”) and non-representative oxygen vacancy concentration and TM oxidation states, due to ill-defined oxygen activity in the material. The latter may be called the “oxygen activity gap” since it refers to the oxygen chemical potential in the oxide. In many cases, the second gap is even more serious than the first one, since surface defects typically equilibrate very quickly (with a rate much higher than oxygen exchange) with the bulk defects,^6,12^ particularly at elevated temperatures.

Not surprisingly, considerable efforts are undertaken to reduce these gap(s) and thus to approach *operando* conditions during chemical surface analysis. In particular, *in situ* or *operando* analytics of the surface chemical properties are of central importance for understanding reaction and degradation mechanisms in more detail. The recent worldwide increase of ambient pressure XPS (AP-XPS) tools partly closes the pressure gap in the case of XPS analysis, but access remains limited to beamtimes at synchrotron facilities for most researchers. Moreover, many other techniques like Auger microscopy or ion-based techniques like secondary ion mass spectroscopy (SIMS) or low-energy ion scattering (LEIS) offer no ambient pressure capability at all.

In this paper we show that we can close the oxygen activity gap in UHV while the pressure gap (of the gas phase) remains. Instead of changing the bulk oxygen chemical potential by providing a gas phase, we modify the oxygen activity in the investigated working electrode (WE) oxide by using a solid oxide cell with an oxygen ion buffering counter electrode, sketched in Fig. 1b. Application of a voltage between the two electrodes moves oxygen ions and changes the oxygen chemical potential *μ*(O_2_)_WE_ with respect to *μ*(O_2_)_CE_ without the need for a gas phase. Recent studies have shown that the surface chemical properties of MIEC oxides (especially transition metal oxidation states and exsolution of metallic species) are in large parts governed by the oxygen activity in the electrode material (which can be modified by the electrode overpotential) rather than the exact atmospheric composition.^12–14^ We may use this tool for tuning the oxygen chemical potential in the bulk of an oxide so that the bulk and surface defect concentrations correspond to those of realistic operational conditions. Accordingly, relevant oxidation states and electrochemical stability window of oxide surfaces can be investigated even in UHV. Although the electrochemical approach for defining oxygen activities in oxides is very well-known in solid state ionics, it was so far hardly applied in surface analytics studies under UHV conditions, and only two publications using much worse performing oxygen buffer electrodes are known to the authors.^15,16^

In this paper, we show that by using a “battery-type” solid oxide cell in UHV, the oxygen activity in the working electrode can indeed be controlled electrochemically simply by adjusting the cell voltage. The key to enabling a well-defined oxygen activity is the usage of an oxygen-ion buffering counter electrode with a Fe/FeO phase mixture that is embedded in a porous Gd-doped ceria (GDC) backbone. Similar concepts with an oxygen deficient single-phase GDC,^16^ or Pd/PdO phase mixture^15^ in the counter electrode were used in literature, but suffered from ill-defined oxygen activity in the counter electrode or fast oxygen loss. The oxygen activity at the Fe/FeO equilibrium in the counter electrode is independent of the Fe : FeO molar ratio, and precisely known from reference measurements in literature.^17^ In this work we show how such a counter electrode can be prepared and preconditioned to achieve reproducible oxygen activity. As proof of concept measurements we explored the surface chemistry of GDC and Fe-doped SrTiO_3_ (STFO) by means of XPS, and compare it to the bulk reducibility that is determined at the same time by coulometry. We restrict ourselves to XPS investigations to proof that these relate well to previously published AP-XPS data. The combination of different UHV-based measurement techniques is part of ongoing work but would be out of scope in this publication where the focus lies on the model cell design and the proof of its applicability, which is independent of the analytic techniques used.

## 2. Electrochemical principles of solid oxide cells in UHV

In this section, we discuss the basic principles of operating a solid oxide cell with an oxide thin film working electrode under UHV conditions. For understanding the implications of operating a cell in UHV, let us first consider the WE of a single chamber SOC in an atmosphere with defined oxygen chemical potential (*μ*(O_2_)_gas_). At open circuit conditions, oxygen exchange reactions will create an equilibrium between *μ*(O_2_)_gas_ and the defect chemistry of the electrodes, so that *μ*(O_2_)_gas_ = *μ*(O_2_)_WE_ = *μ*(O_2_)_CE_. An applied voltage *U*_cell_ is then the sum of WE, CE and electrolyte overpotentials, given by

1 *U*_cell_ = *η*_WE_ + *η*_CE_ + *I*_DC_*R*_YSZ_.

For simplicity, we assume a working electrode in which the surface reaction governs the oxygen exchange kinetics, and overpotentials due to electron or ion conduction within the WE are negligible. Such a situation is often found for thin film electrodes.^6,7,18^ In such a case, *μ*(O_2_)_WE_ = *μ*(O_2_)_gas_ + 4*Fη*_WE_. Moreover, we neglect overpotential losses in the CE, so that *μ*(O_2_)_CE_ = *μ*(O_2_)_gas_ (meaning fast kinetics). In that case, the cell voltage is given by2
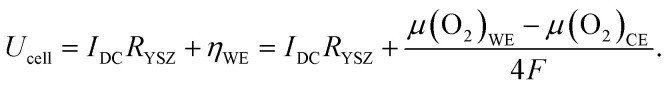
In such a cell, the DC current I_DC_ is proportional to the rate of oxygen migration between WE and CE.

In UHV, the situation is more straightforward. Due to the virtual absence of gas phase molecules, oxygen cannot be incorporated, and O_2_ is not released at a relevant rate when the oxygen activity in WE and CE is sufficiently low. O_2_ release will be discussed in detail later in this section. In such conditions, the cell behaves like an oxygen ion battery with two mixed conducting electrodes with electrochemically controllable oxygen content. This concept has also been recently proposed for energy storage.^19^ When the cell equilibrates at a constant voltage, the DC current and thereby, any ion or electron conduction overpotential (including conduction within the electrode and across interfaces) is zero. Still, a change of the cell voltage (within reasonable limits) causes a finite amount of oxygen ions to exchange between WE and CE until their oxygen stoichiometries reach a new equilibrium. This voltage is then an equilibrium voltage called *U*^eq^_cell_. Quantitatively, this equilibrium is EXACTly given by3
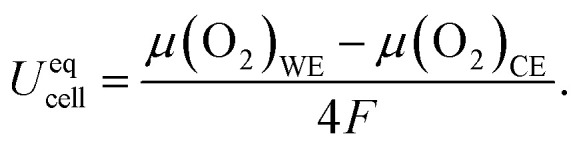


Although eqn (2) and (3) give the impression that *U*^eq^_cell_ equals *η*_WE_, these two equations describe two fundamentally different cases. *η*_WE_ is the kinetic overpotential of a reaction with an OCV of zero, whereas *U*^eq^_cell_ is the state-of-charge dependent OCV of an oxygen ion battery.

### 2.1. Electrochemical oxygen activity control (EXACT)

Since chemical potential *μ* and activity *a* are linked by *μ* = *μ*^0^ + *RT* ln(*a*), eqn (3) can also be expressed in the form4
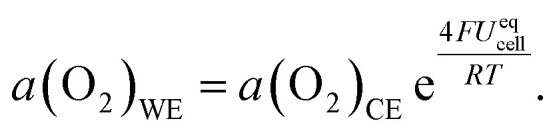


Consequently, the key to controlling the oxygen activity in the WE is achieving a known oxygen activity in the CE while remaining in UHV. An oxygen-deficient MIEC counter electrode would have a highly stoichiometry-dependent oxygen activity. Hence, in this study we use a Fe/FeO phase mixture which provides a constant and known oxygen activity that is determined by the free enthalpy of formation Δ_f_*G*^0^ of FeO. This value is known from literature^17^ by

5 Δ_f_*G*^0^(FeO) = −303 097 + 683.498*T* − 91.3978*T* ln *T* + 0.05180*T*^2^ [J mol^−1^].

We can use this expression to replace *a*(O_2_)_CE_ in eqn (4) and get the final form6
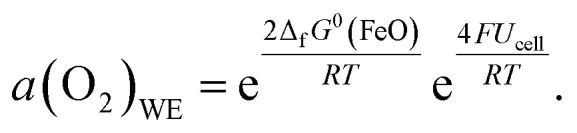


**Fig. 2:**
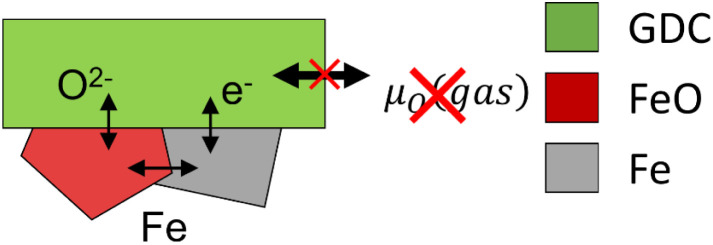
Ion/electron exchange reactions that couple the Fe/FeO phase equilibrium to the oxygen chemical potential in the GDC backbone of the counter electrode.

**Fig. 3:**
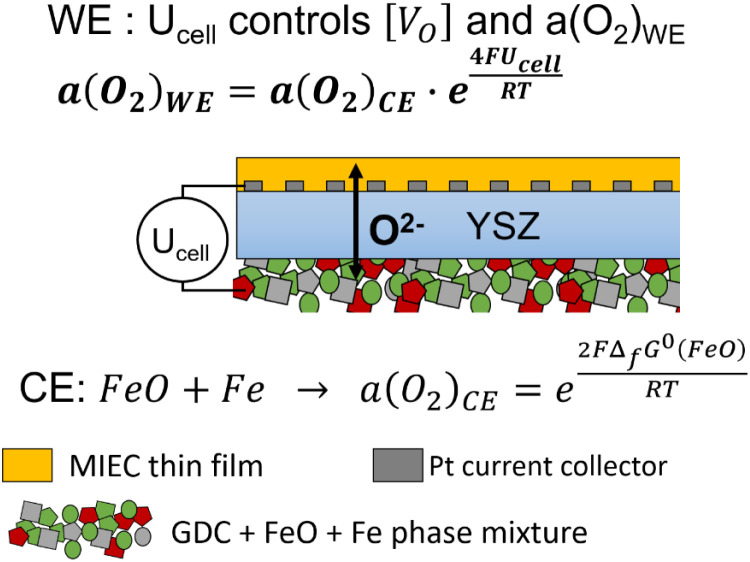
Sketched principle of the working electrode oxygen activity control in the EXACT model cell in UHV.

However, coupling the defined oxygen activity of the metal-oxide phase equilibrium to an oxygen ion conducting electrolyte without a gas phase is experimentally not trivial. Oxidation and reduction of Fe must occur by means of electron and oxygen ion transfer into an electrode backbone material that is electronically connected to a current collector and also enables reversible O^2−^ ion transport into the YSZ electrolyte. Furthermore, this backbone should mechanically stabilise the Fe/FeO particles that undergo significant chemical expansion. Fig. 2 shows a sketch of the ion/electron exchange reactions in the counter electrode, although some mechanistic details such as exact Fe and O diffusion pathways are not fully understood. GDC was chosen as the MIEC backbone material due to its chemical stability and mixed conduction under reducing conditions. As oxygen storage medium, Fe|FeO was selected because of its fast oxidation/reduction kinetics that are attributed to its high concentration of Fe vacancies, resulting in a stoichiometry of Fe_0.95_O at the phase equilibrium.^20^ Moreover, the oxygen activity at the phase equilibrium is in the range where the GDC backbone becomes a mixed conducting material, so that the electron transfer is sufficiently fast. The electrochemical key principles for oxygen activity control in UHV are sketched in Fig. 3.

### 2.2. Upper oxygen activity limit by O_2_ release

In UHV, oxygen incorporation is virtually impossible, but O_2_ release is still possible, which may lead to two problems. Firstly, kinetic overpotentials are no longer negligible in case of a substantial O_2_ release rate. Secondly, only a limited amount of oxygen ions is stored in the CE, leading to limited time for experimentation. For oxygen activities corresponding to fuel electrode materials, the effective *p*(O_2_) is many orders of magnitude below the chamber pressure so that the O_2_ release rate is virtually zero.

The oxygen release rate can be predicted for oxygen electrodes from the kinetics of oxyen exchange in O_2_ atmosphere. The Equilibrium O_2_ exchange rate 
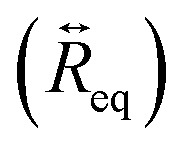
 of oxygen is coupled to the *p*O_2_ and temperature-dependent tracer exchange coefficient k*, or area-specific resistance (ASR) of the electrode by the relation^21,22^7
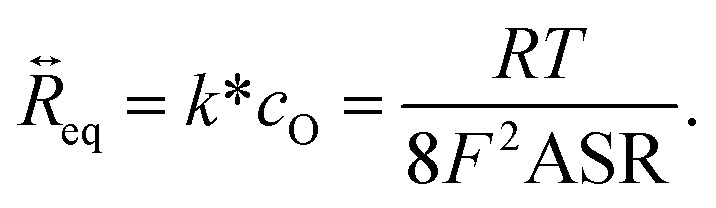
Therein, *c*_O_ is the lattice oxygen concentration (mol cm^−3^), *R*, *T* and *F* are the gas constant, Faraday constant and temperature, respectively. 
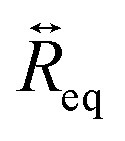
 is the molar flux of O_2_ per cm^2^ per s that is incorporated and released at the same rate. When this material is immediately transferred into UHV 
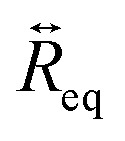
 will equal the O_2_ release rate. A typical counter electrode contains roughly 10^−5^ mol Fe cm^−2^. For typical conditions (600 °C), the time for which oxygen can be provided from the CE is calculated either from the ASR, or the electrical oxygen release current

8 Δ*t* ≈ 53 s Ω^−1^ cm^−2^·ASR.

To put this in perspective, an O_2_ release rate of 10^−10^ mol cm^−2^ s^−1^, or 38 μA cm^−2^ corresponds to an ASR of 975 Ω cm^2^. With this release rate, the CE reservoir lasts for *ca.* 14 hours, which is a reasonable timescale for conducting surface science experiments. A (thin film) air electrode that has an ASR of 10 Ω cm^2^ in 1 bar O_2_ and a scaling of ASR ∝ *p*O_2_^−0.5^ will approach this value at *ca. a*(O_2_) = 0.1 mbar. At lower temperature, oxygen activity values equivalent to 1 bar or more are achievable, as shown in the SI.

### 2.3. Thin film coulometry

When changing the cell voltage, a new oxygen chemical potential becomes established in the working electrode *via* changing the corresponding oxygen content and thus the oxygen defect concentration. We can get information on the stoichiometry change by measuring the charge required to establish the new composition. This approach is known as coulometric titration.^23^ In our solid oxide cell, almost the entire charge passing the electrolyte corresponds to motion of oxygen anions since the ionic transference number of YSZ is very close to 1 under typical conditions.^23^ When no oxygen exchange at the WE surface occurs, the integral charge is proportional to changes in the oxygen content in working and counter electrodes. The corresponding oxygen stoichiometry variations can be calculated by9
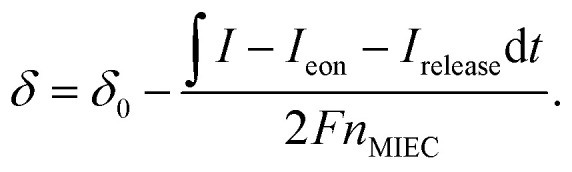


Therein, *δ* is the oxygen deficiency, *δ*_0_ is the oxygen deficiency at the start of the electrochemical experiments, *n*_MIEC_ is the number of MIEC formula units in the WE thin film and *I*_eon_ is the leak current caused by the electronic conduction in the electrolyte, and *I*_release_ the electrical current carried by O_2_ release. At oxygen activities that are typical for fuel electrodes (equivalent to <10^−15^ bar), O_2_ release is negligible. However, especially under strongly reducing conditions, typically *U*_cell_ < −0.5 V, electronic conduction in the electrolyte^23,24^ can become a relevant source of charge leakage, but it's magnitude is known from electronic conductivity measurements of YSZ in literature, and given by10
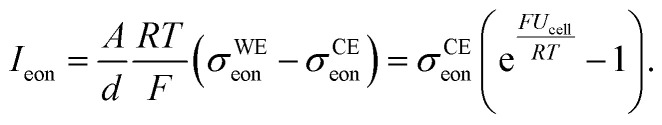


Therein, *A* and *d* are the cell area and electrolyte thickness, respectively and *σ*^CE^_eon_ is the electronic conductivity of YSZ at the oxygen activity of the counter electrode. This value can be either taken from literature,^24^ or empirically optimised so that the charge after a reduction-oxidation cycle converges towards zero. Currents due to O_2_ incorporation from the residual gas may be another relevant error source, but only in case of “high” vacuum chamber pressure above 10^−8^ mbar.

## 3. Experimental

### 3.1. Counter electrode fabrication

In the first step, 80 wt% Ce_0.9_Gd_0.1_O_1.95_ (270 nm mean particle size, Treibacher, Austria) and 20 wt% Fe_2_O_3_ nanoparticles were mixed with organic ink vehicle (Fuelcellmaterials) in a 1 : 1 weight ratio and homogenised by ball milling. This paste was then spin-coated onto the rough side of single-side polished 10 × 10 × 0.5 mm^3^ [100] oriented YSZ single crystals (Crystek) at a speed of 2400 rpm. After drying, Pt paste (Tanaka TR-7907) as a current collector was brushed onto the spin-coated layer, and the electrode was sintered at 1050 °C for three hours in air with 3 °C min^−1^ heating/cooling rates. The resulting counter-electrode had a thickness of roughly 14 μm. One symmetrical cell with counter electrodes on both sides was fabricated to characterise oxide ion storage in these counter electrodes. The Fe/FeO phase mixture required for a stable *a*(O_2_)_WE_ was electrochemically generated during a conditioning step inside the analysis UHV chamber; see results and discussion.

### 3.2. Determination of Fe content in the counter electrode

The total amount of iron in the symmetrical cell was determined using ICP-OES after wet acid digestion. The iron oxide from the symmetrical GDC/Fe_2_O_3_ cell was dissolved in HCl and diluted to a volume of 10 mL. The measurement was performed on an Agilent 5110 ICP-OES equipped with a cyclonical spray chamber and a CETAC ASX-520 autosampler. Signals were quantified by an external calibration with matrix-adjusted dilutions of certified reference materials. The Fe line at 259.940 nm was used for quantification, Fe line at 234.350 nm for quality control.

### 3.3. WE thin film deposition

After sintering of the counter electrode, a 5/100 nm thick Ti/Pt current collector was deposited on the polished side of the YSZ crystals. The Pt film was microstructured by photolithography and ion-beam etching into a grid with 5 μm stripes and 25 × 25 μm holes for minimising electron conduction overpotentials in the WE.

As working electrode material, fluorite type Ce_0.9_Gd_0.1_O_1.95_ (GDC10) and perovskite-type SrTi_0.6_Fe_0.4_O_3−*δ*_ (STF100), as well as the 5% A-site sub-stoichiometric material Sr_0.95_Ti_0.6_Fe_0.4_O_3−*δ*_ (STF95) were used. The GDC10 target was uniaxially pressed in a 30 mm die from commercially available powder (Treibacher, Austria) and sintered at 1500 °C. STF100 and STF95 were synthesised by solid-state synthesis of appropriately weighed SrCO_3_, Fe_2_O_3_ and TiO_2_ homogenised in a ball mill and uniaxially pressed in a 30 mm die. The resulting pellets were calcined at 1150 °C for three hours and subsequently sintered at 1350 °C for five hous.

From the polycrystalline targets, thin films were deposited by pulsed laser deposition using a 248 nm pulsed excimer laser with an energy of 100–110 mJ (on the target) with 10 Hz pulse frequency. The substrate was heated to 600 °C at a distance of 6 cm and rotated with 1 rpm for homogeneous deposition. Both films were deposited for 30 minutes. The resulting films were polycrystalline with a thickness of 330 nm for STFO and 520 nm for GDC.

### 3.4. XPS measurement

**Fig. 4:**
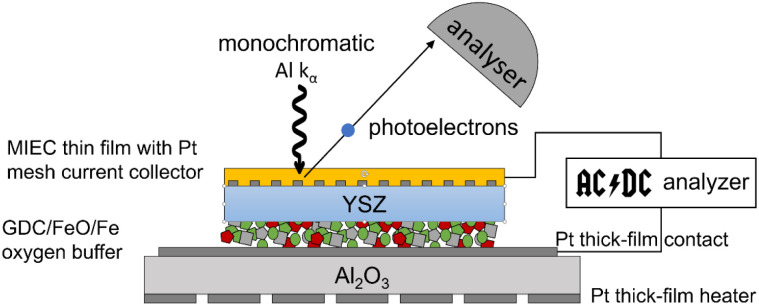
Sketch of the combined electrochemical and XPS investigations of the EXACT model cell.

After cell fabrication, the cells were fractured into 5 × 5 × 0.5 mm^3^ pieces and inserted into a PHI Versaprobe 3 XPS spectrometer with a base pressure of 3 × 10^−10^ mbar. The sample was mounted on a custom resistive heater consisting of a Pt–GDC composite thick film meander on a 1 mm thick Al_2_O_3_ plate capable of heating the sample to 800 °C. Electrical contact was established by conductive Pt coating of the heater (for the CE) and Pt–Ir alloy needles for the WE. These Pt–Ir needles also served as mechanical sample mounts, as sketched in Fig. 4. Electrochemical measurements were carried out using a Bio-Logic SP-200 electrochemical test station capable of potentiostatic, galvanostatic and impedance-based measurement techniques. The sample temperature was determined using impedance spectroscopy, from which the electrolyte conductivity was calculated and compared to reference measurements carried out in a homogeneously heated furnace.^25^

After heating the model cell to 400–650 °C (depending on sample type), a counter electrode “conditioning” step was carried out in which a constant current of ∼1 mA cm^−2^ was applied for 1200 s, during which O_2_ was released from the MIEC WE. In the CE Fe_2_O_3_ was reduced to a mixture of Fe and FeO.

XPS spectra were acquired using a monochromated Al k-α X-ray source with 50 W power and 200 μm spot size. Photoelectrons were collected at a 45° angle relative to the surface normal at an analyser pass energy of 27 eV for detail scans and 140 eV for survey spectra. The spectra were fitted and evaluated using CasaXPS software. For quantification, a mean-free path exponent of 0.75. Empirically derived cross-sections from reference compounds by Brundle and Christ^26^ were used and further corrected for the asymmetry factor stemming from the 45° source-analyser angle.

## 4. Results and discussion

### 4.1. *In situ* generation of Fe/FeO mixtures in the CE

**Fig. 5:**
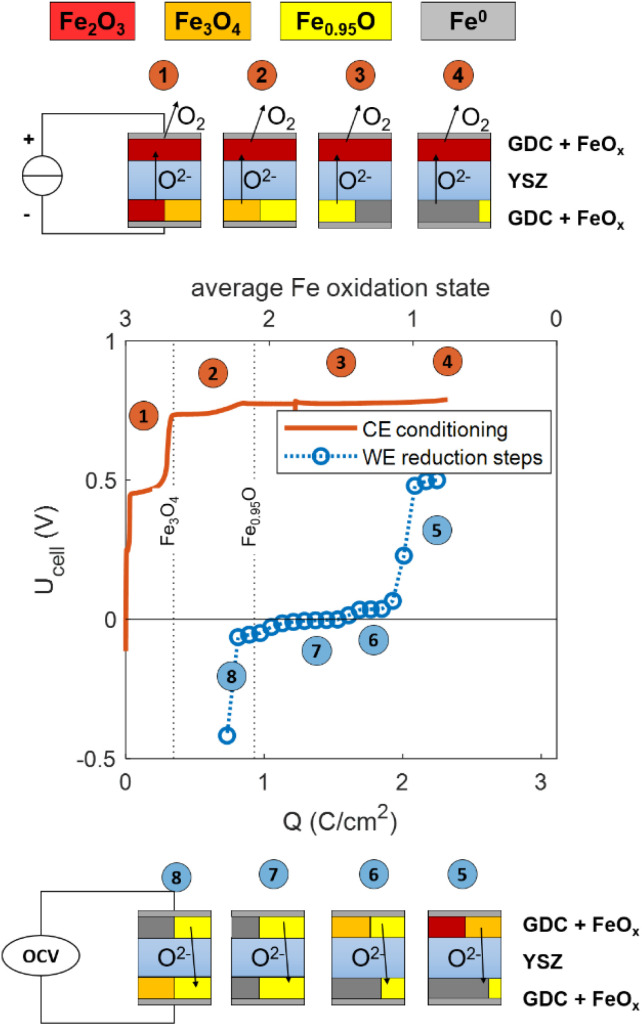
Electrochemical characterisation of a symmetrical cell with two GDC + FeO_*x*_ electrodes at 650 °C un UHV. The orange line shows the conditioning step, and the blue symbols represent the cell OCV after subsequent galvanostatic steps with the negative current. The sketches represent the different phase equilibria at the top and bottom electrodes during CE conditioning (top) and stepwise reduction of the upper electrode (bottom).

After fabrication, the CE consists of GDC and Fe_2_O_3_. The oxygen buffering Fe/FeO phase mixture was generated *in situ* by applying a positive current by an initial galvanostatic conditioning step. To characterize this in more detail, a symmetrical cell with GDC/Fe_2_O_3_ electrodes on both sides was fabricated and heated in UHV to 650 °C. In the following discussion, the upper electrode will be called the working electrode (WE), while the other will function as the counter electrode (CE). The voltage *vs.* charge curves of this cell are given in Fig. 5. The lower *x*-axis shows the total charge that passes the cell, and the upper *x*-axis gives the calculated average Fe^*x*+^ oxidation state in the CE. This is linked to the charge axis by the relation *x* = 3 − *Q*/(*Fn*(Fe)), where *Q* is the charge and *n*(Fe) molar amount of Fe in the CE, determined by ICP-OES, see experimental section. The orange line shows the initial galvanostatic conditioning step, during which a constant positive current of 1 mA cm^−2^ is applied. This process causes O^2−^ ions to move from the CE to the WE. These cannot be stored in the WE, which is oxidised after fabrication. Therefore, O_2_ is released from the WE into the vacuum. During this process, the chamber pressure rises from ∼10^−9^ mbar into the medium 10^−8^ mbar range. In the CE, O^2−^ is removed from the Fe_2_O_3_ phase. Clearly visible, the voltage increases in slightly blurred steps, that correspond to the different iron oxide phase equilibria in the CE. (Fe_2_O_3_ → Fe_3_O_4_ → Fe_0.95_O → Fe). The Fe oxide phase boundaries are plotted as dashed lines and the CE phase equilibrium at each plateau is sketched according to the markers 1–4. A total charge of 0.8 C cm^−2^ is required to reach a plateau at ∼0.8 V, which indicates Fe/FeO phase equilibrium in the CE. After reaching 2 C cm^−2^, there is a slight increase in voltage, indicating that most FeO is consumed, and the conditioning was stopped after reaching an average Fe oxidation state of 0.5 presumably since not all Fe is electrochemically accessible with fast kinetics.

**Fig. 6:**
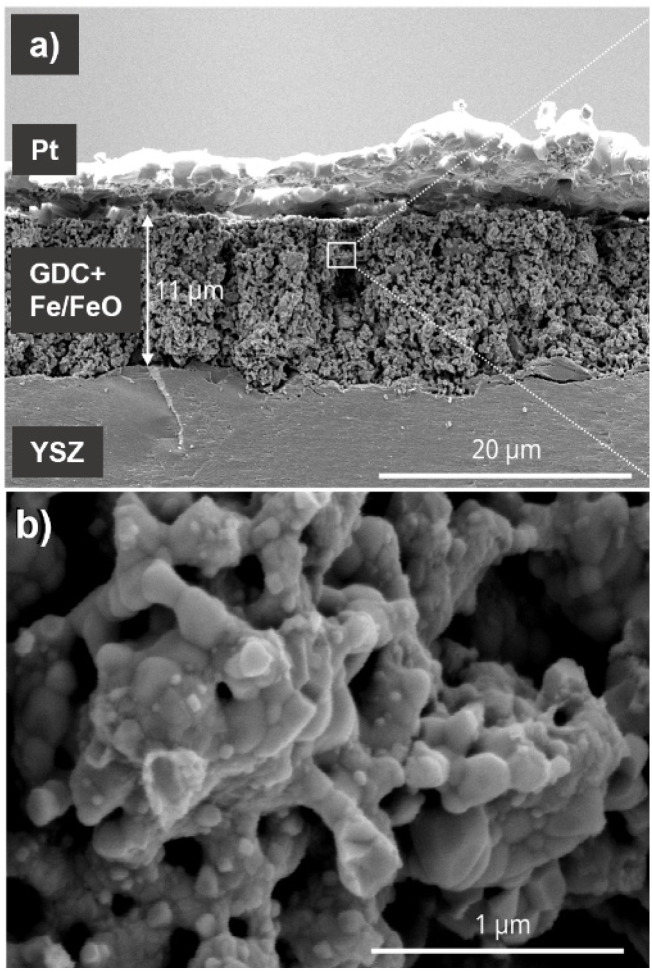
Secondary electron image of a cleaved cross section of the symmetric porous electrode after electrochemical characterization in UHV (reduced after measurement). (a) 8k magnification (b) 116k magnification.

To show that the Fe/FeO equilibrium gives a reliable reference oxygen activity over a sufficiently large charge range, stepwise galvanostatic coulometry was performed after the conditioning of the CE. To that end, negative current pulses of −0.5 mA cm^−2^ were applied for 200 s (Δ*Q* = −0.1 C cm^−2^). These were followed by resting steps of 300 s (to allow for equilibration of the sample), after which the OCV was measured. These OCV values are plotted as blue markers in Fig. 5. The voltages follow the different iron oxide phase equilibria in the WE (Fe_2_O_3_/Fe_3_O_4_ for 2.3–2 C cm^−2^ and FeO/Fe_2_O_3_ for 2–1.5 C cm^−2^) *versus* the Fe/FeO phase equilibrium in the CE. In the charge range of 1.5 to 1 C cm^−2^, the equilibrium voltage is below 10 mV, as both electrodes are in the Fe/FeO phase equilibrium. Below 1 C cm^−2^, the cell voltage becomes negative because all Fe in the CE is oxidised (resulting in a FeO/Fe_3_O_4_ phase mixture), while the WE still has the Fe/FeO phase mixture. SEM images of the CE after the measurement sequence in Fig. 6 show that the GDC backbone remains stable, and no cracking or delamination occurs due to chemical expansion. Due to their small size and similar morphologies, a clear differentiation between FeO_*x*_ and GDC particles is not possible from the SEM data. In summary, within a (total) charge range of 1–2.2 C cm^−2^, *a*(O_2_)_CE_ remains at the Fe/FeO equilibrium, so a charge of 1.2 F cm^−2^ (almost 10^19^ O^2−^ ions per cm^2^) can pass through the cell without changing the oxygen chemical potential of the CE. In comparison, the charge required to reduce or oxidise the thin-film working electrode is at least one order of magnitude smaller, so the CE buffer capacity is easily sufficient. Due to the usage of UHV, also any oxygen incorporation from the residual gas is extremely slow. Typically, the equilibrium DC current during a constant voltage step is <10^−8^ A. Assuming that this current stems from reactions with the residual gas, a depletion of the CE buffer would require >10^8^ s, or more than one year. Experimentally, the CE stability was monitored for 10 hours, see SI, Fig. S8. Further verification that *a*(O_2_)^CE^ remains at the Fe/FeO equilibrium was demonstrated by CV sweep on a STFO thin film that contained FeO_*x*_ impurities after fabrication, see SI for details. Even more reliable verification of the CE oxygen activity might be gained by adding a properly placed Fe + FeO containing reference electrode, with geometries suggested in literature.^27,28^ Due to a lack of spare electrical feedthroughs in the used XPS apparatus, this could not be performed for the shown experiments. Nonetheless, the charge during the conditioning step needs to be chosen carefully. Too little charge will let the CE rest at the Fe_3_O_4_/FeO equilibrium, which has a potential *ca.* 50 mV above Fe/FeO equilibrium. With too much charge, all Fe becomes metallic and the nonstoichiometry of the GDC backbone becomes the ion buffering mechanism, at a voltage slightly below the Fe/FeO equilibrium.

### 4.2. Oxygen surface chemistry of a Ce_0.9_Gd_0.1_O_1.95−*δ*_ thin film electrode

After verifying that the oxygen activity in the CE is indeed stable and known, a model cell with a GDC thin film working electrode and oxygen ion buffering CE was investigated, see experimental section for details.

Acceptor doped ceria is probably the MIEC with the best studied surface chemistry. Its simple binary oxide structure makes the surface less prone to cation segregation^29,30^ and surface reconstructions in comparison to perovskite-type oxides. Many studies have shown enhanced surface reducibility of GDC with different techniques, including thermogravimetry,^31^ chemical capacitance^32^ and ambient pressure XPS.^11,33,34^ As explained in the introduction, the “EXACT” method allows for simple coulometric determination of the working electrode bulk oxygen stoichiometry, and XPS allows for precise measurements of the degree of surface reduction by measuring the concentration of Ce^3+^, which has a signature in Ce3d, Ce4d and Ce4f states.^11^ Most ambient pressure XPS studies in the literature were carried out in various H_2_/H_2_O gas mixtures^14,33–36^ or CO/CO_2_ mixtures,^14,37,38^ in which the GDC surface contains numerous hydroxyl or carbonate groups, which are not present in the UHV studies shown in the following.

**Fig. 7:**
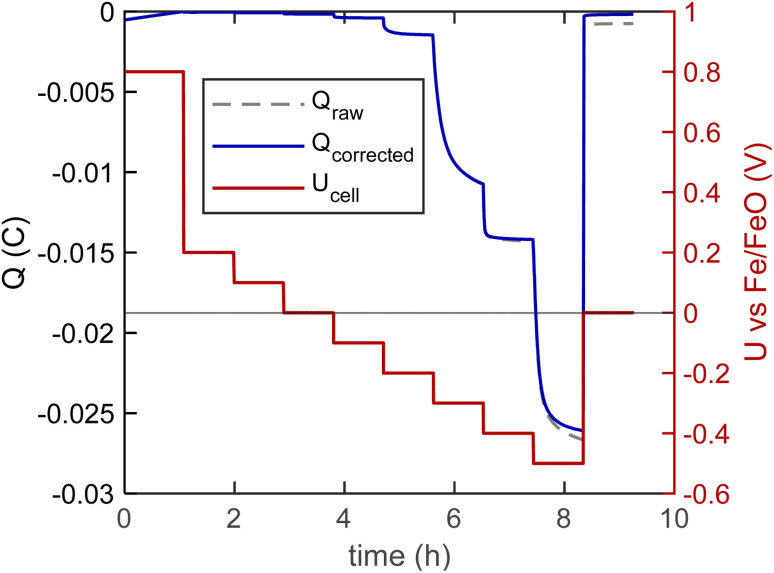
Voltage step curve (red line), measured charge (dashed gray) and leakage-current corrected charge (blue) during XPS acquisition of the GDC10 sample at 510 °C.

A model cell with a 520 nm thick GDC10 working electrode was heated in the XPS spectrometer, and XPS data acquisition was started after counter electrode conditioning, while changing the cell voltage stepwise in the sequence visible in Fig. 7 red curve. The dashed grey and solid blue curves (*Q*_raw_ and *Q*_corrected_, left *Y*-axis) show the electrical charge flowing during the experiment. For the curve *Q*_corrected_, electronic leakage through the electrolyte was subtracted according to eqn (10). The effect of this electronic leakage correction is hardly noticeable for the given data, but it is applied here as an example, since it might be necessary if a larger cell voltage window is used. This *Q*_corrected_ was then used to determine the bulk oxygen deficiency in GDC by using eqn (9). As a stoichiometry reference, we assume 100% Ce^4+^, corresponding to Ce_0.9_Gd_0.1_O_1.95_ at +800 mV (*a*(O_2_) = 7 × 10^−8^ bar). Subtly visible, at +800 mV, the charge curve is slightly turning upward, due to a small O_2_ release rate, corresponding to a current of 130 nA (3 × 10^−13^ mol O_2_ s^−1^). The CE oxygen storage capacity at this O_2_ loss rate would still last for roughly 20 days.

**Fig. 8:**
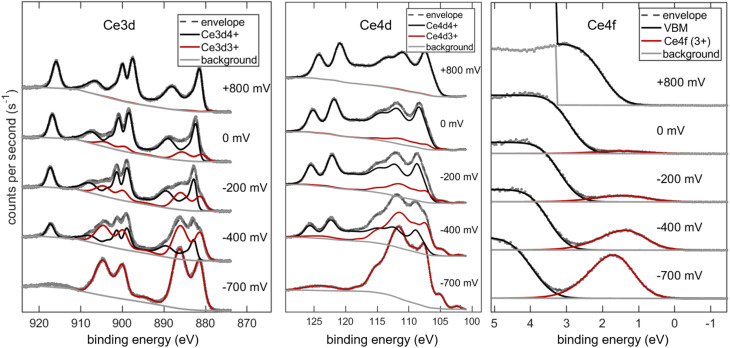
XPS spectra of the Ce3d, Ce4d and Ce4f (valence band) regions of the GDC sample at 510 °C and various applied cell voltages.

At the same time, XPS detail spectra of the Ce3d, Ce4d, Ce4f (valence band) and O1s regions were acquired and fitted, shown in Fig. 8 and S3 (for O1s). The XPS spectra at −700 mV were obtained after the voltage program, in order to have a spectral reference of pure Ce^3+^. Due to the different photoelectron energies of the three transitions, the effective IMFP varies significantly. By considering the 45° photoelectron emission angle and using the TPP-2M equation,^39^ we calculate effective attenuation length (EAL) values of 0.85 nm, 1.55 nm and 1.65 nm for Ce 3d, Ce4d and Ce4f, respectively.

The structure of Ce3d and Ce4d peaks is extremely complex, containing several satellite peaks in addition to the spin–orbit splitting. The origin of this complex peak shape is discussed in the literature.^40^ Here, we simply treat the spectra as a linear combination of Ce^4+^ and Ce^3+^ reference spectra, that were acquired at +800 mV and −700 mV, respectively. Consequently, only three independent parameters (peak position and intensity of Ce^3+^ and Ce^4+^ reference patterns were used for fitting the Ce^3+^ fraction for the spectra at intermediate potentials. Reduction of ceria is also clearly visible in the valence band region, in which the VBM (black curve) is primarily of O2p character, while the peak around 1.5 eV binding energy (red) corresponds to the additional electron in Ce^3+^, which is a Ce4f state.^36^ For quantitative calibration, the Ce4f intensity in the spectrum at −700 mV was taken as the 100% Ce^3+^ reference. Also visible, the position of the valence band maximum shifts to higher binding energies upon reduction of the sample. This is due to a change of the Fermi energy within the band gap, and is known from literature.^7,14,35^ A Fermi energy shift close to 1 eV V^−1^ cell bias was observed (as expected) and is plotted in the SI. XPS data and peak models are available as CasaXPS Vamas files provided as supplementary files.

**Fig. 9:**
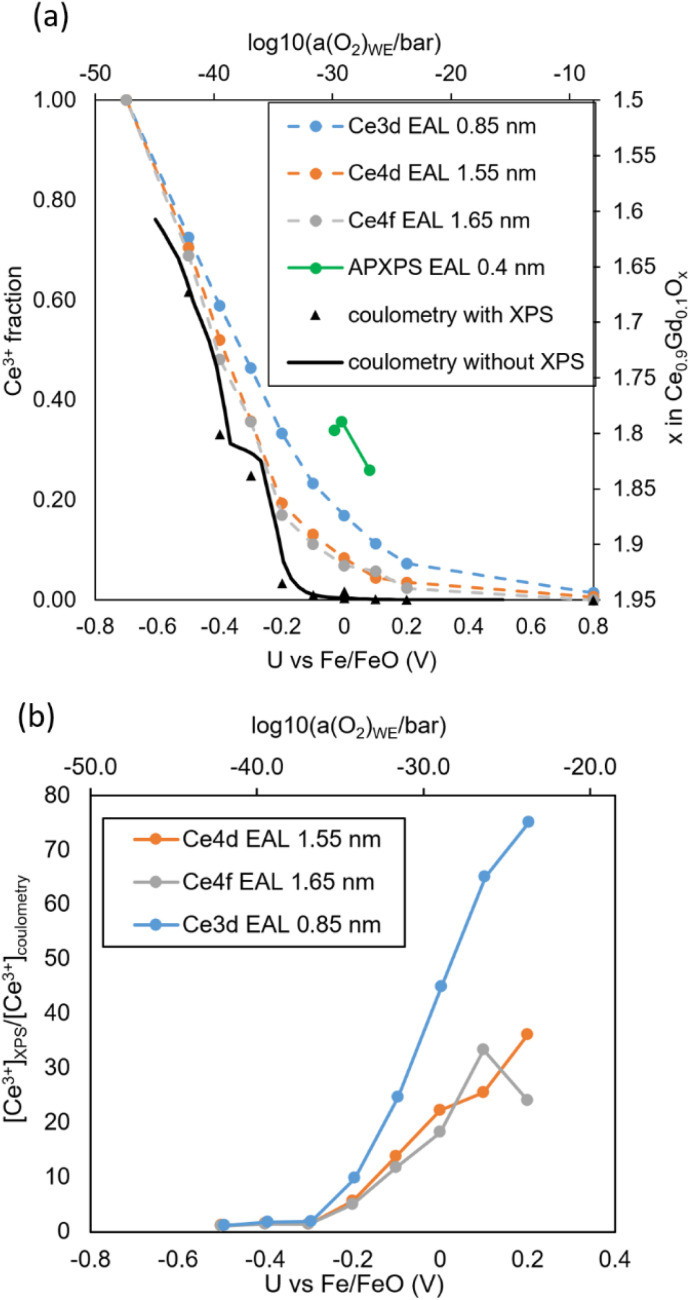
(a) Ce^3+^ fraction (left *y* axis) and oxygen stoichiometry (right *y* axis) of the GDC10 film, determined by XPS (symbols) and bulk coulometry (black line) as function of cell voltage (lower *x*-axis) at 510 °C. The corresponding *a*(O_2_)_WE_ values are given in the upper *x*-axis. The green data series was taken from synchrotron-based ambient-pressure XPS measurements in literature.^11^ (b) Ratio of surface and bulk Ce^3+^ fraction.

All fitted Ce^3+^ fractions are combined in Fig. 9a. Therein, the Ce^3+^ fraction could be determined from three different XPS peaks with varying surface sensitivity as well as coulometry, which reflects the average bulk reducibility. Two sets of coulometry data are shown: Black triangles correspond to the DC data shown in Fig. 7 (during XPS acquisition), and the black solid line was measured with a voltage step size of 25 mV for better resolution without parallel XPS acquisition. In addition, Ce^3+^ fractions from ambient pressure XPS data on 20 mol% Sm-doped ceria published by Chueh *et al.*^11^ (green data series) is given. Due to the tuneable synchrotron light source used in the cited study, the EAL is 0.4 nm for Ce4f photoelectrons. The two *x*-axes of the plot are linked by eqn (6). The two *y*-axes are linked by the stoichiometry of reduced GDC, following the formula Ce_*x*_^3+^Ce_0.9−*x*_^4+^Gd_0.1_O_1.95−*x*/2._

All XPS data show strongly enhanced Ce^3+^ concentration compared with the bulk coulometry data, which shows noticeable Ce^3+^ fractions only below −100 mV. Accordingly, the measurements confirm the easier surface reducibility, compared to bulk. The extent of preferential surface reducibility is shown in Fig. 9b, where the ratio of Ce^3+^ concentration determined by XPS (surface) and coulometry (bulk) data ([Ce^3+^]_XPS_/[Ce^3+^]_bulk_) is plotted.

Clearly visible, the [Ce^3+^] surface enhancement is the highest for the most surface sensitive XPS peak (Ce3d), and weakly reducing conditions (+0.2 V *vs.* Fe/FeO). The most surface sensitive XPS peak (Ce3d) shows twice as much Ce^3+^ as the Ce4d and Ce4f peaks, while having only halve of the EAL. This shows that it is really the topmost 1–2 atomic layers (0.2–0.4 nm) that are preferentially reduced. Green symbols in Fig. 9a were taken from ambient pressure XPS measurements in literature,^11^ and show considerably higher Ce^3+^ concentration. However, this study used tuneable synchrotron radiation (green), with an effective EAL of only 0.4 nm, giving a very good quantitative agreement with our UHV studies. This also indicates that the enhanced surface reducibility of GDC is not due to hydroxyl groups that are present in H_2_ + H_2_O mixtures,^14^ but an intrinsic property of the GDC surface. Accordingly, comparison of such measurements with a closed oxygen activity gap and NAP-XPS measurements yields even additional mechanistic information. Moreover, the comparison also demonstrates that the EXACT method allows a much broader tunability of the oxygen activity, compared to gas mixtures. In literature it is argued, that any broken symmetry around the Ce ion will lift the degeneracy of Ce4f orbitals.^36^ In this case, one of the orbitals decreases in energy and thereby increases the reducibility. This model explains the enhanced reducibility found under compressive and tensile strain,^36^ due to doping with smaller Zr^4+^ or Hf^4+^ ions,^41^ and of course at surface terminations. An additional interesting feature is the “kink” found in the coulometry curve in Fig. 9 around −0.4 V or 10^−40^ bar. This feature is most likely related to an intermediate oxygen vacancy ordered phase that is forming under these extremely reducing conditions, and is part of current investigations with complementary X-ray diffractometry. The appearance of an additional O1s species (shown in the SI) under strong cathodic bias further indicates some kind of structural ordering with two different oxygen sites, such as the hexagonal bixbyite structure.^42^ However, the available data is only an indication and not a proof for vacancy ordered phases. Although vacancy ordering in CeO_*x*_ has been reported in phase diagrams,^43^ its precise effect on the bulk reducibility has not been broadly discussed in literature, because the corresponding oxygen activity is too low to be realised in controlled gas mixtures.

In summary, we could reproduce the enhanced surface reducibility of GDC, and found excellent quantitative agreement with ambient pressure XPS measurements in literature. These results demonstrate that oxygen activity control is possible in UHV and that the resulting surface chemistry is indeed closely related to real cell operating conditions. In comparison with gas mixtures, an even much broader and continuous oxygen activity range can be realised.

### 4.3. Surface chemistry and bulk reducibility of STFO thin films

Also, EXACT model cells with Fe-doped SrTiO_3_ (STF) thin films as the working electrodes were investigated. Two slightly different compositions were chosen: the A-site stoichiometric formula SrTi_0.6_Fe_0.4_O_3−*δ*_ (STF100) and the 5% A-site sub-stoichiometric material Sr_0.95_Ti_0.6_Fe_0.4_O_3−*δ*_ (STF95). Compared to the binary fluorite oxide ceria, the surface structure of perovskite-type oxides is much more complex, due to segregation of A-site oxides, primarily in oxidizing environments, which is summarised in review articles, *e.g.*Ref. 30 and 44. In reducing conditions, B-site transition metals (in our case Fe) may exsolve in the form of metallic nanoparticles.^7,45^ Furthermore, the tendency for exsolution strongly depends on the A : B cation stoichiometry.^46^ Consequently, the surface chemistry of perovskite-type oxides depends not only on stoichiometry, temperature and *p*O_2_, but also on previous annealing conditions – even in the absence of impurities.

**Fig. 10:**
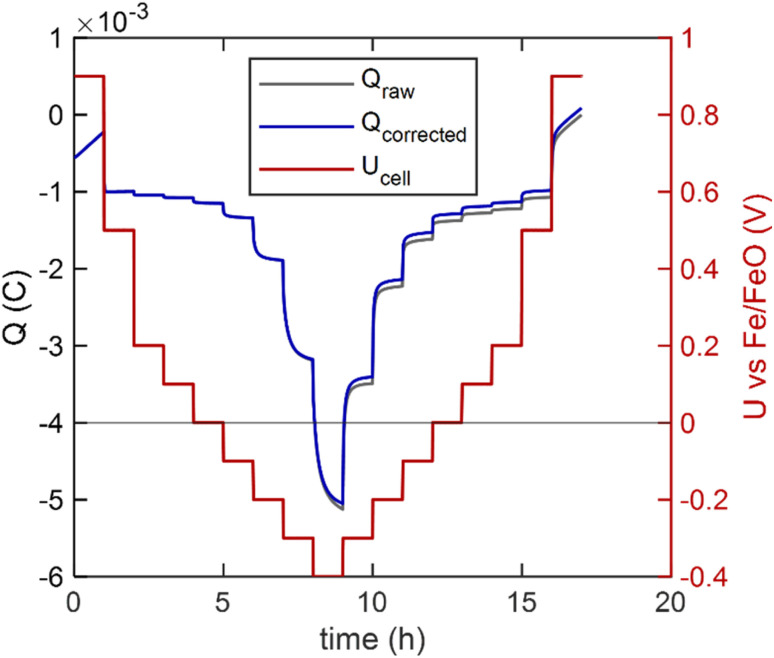
Electrical characterisation of the STF95 sample with parallel XPS acquisition.

In this study, we want to focus on the reversible changes in the STF surface chemistry due to variable oxygen activity. Therefore, we selected a low temperature of 400 °C, at which cation segregation effects are very slow, but oxygen vacancies are still mobile. XPS data of both samples was acquired during the voltage program plotted in Fig. 10 (shown for STF95). At each voltage step, O1s, Ti2p, Fe2p, Fe3p, Sr3d and valence band spectra were acquired. A symmetric reduction-oxidation program was chosen in order to identify possible irreversible effects. Similarly to the GDC10 sample, a slight O_2_ release rate leads to a positive slope in the charge curves at +0.9 V, or *a*(O_2_)_WE_ = 10^−7^ bar. The correction for electronic leakage current (difference between *Q*_raw_ and *Q*_corrected_) only has a marginal effect. The coulometric titration curve of STF100 is almost identical and shown in the SI.

**Fig. 11:**
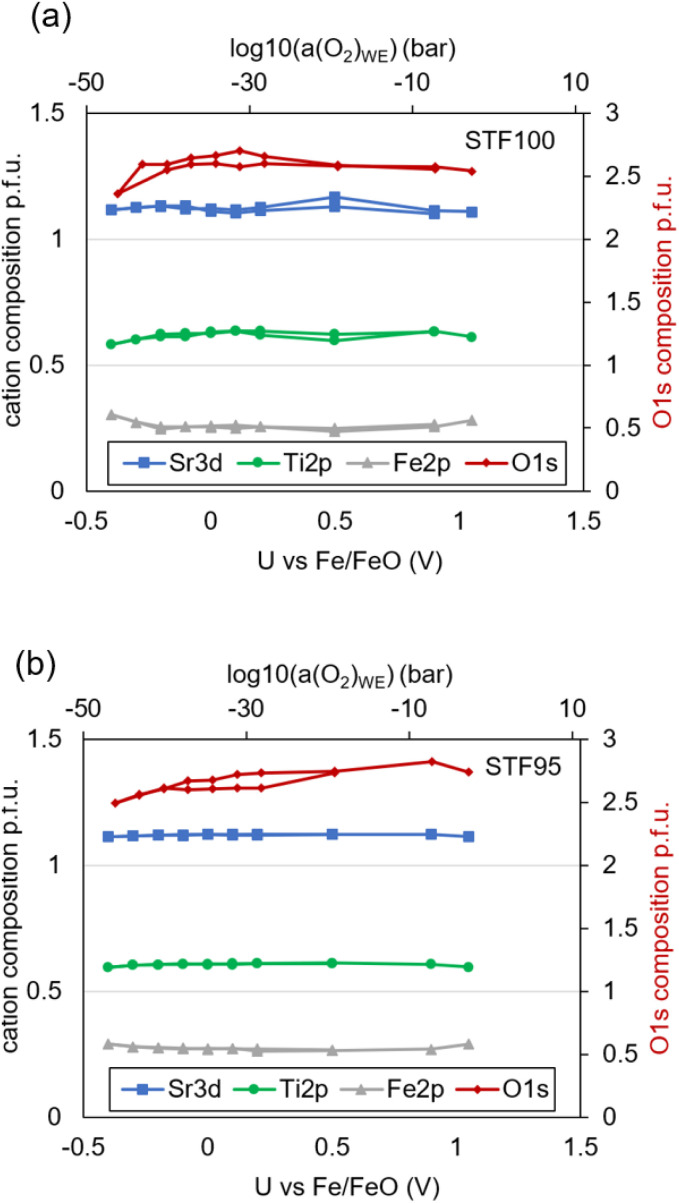
Surface cation composition as function of cell bias at 400 °C determined by XPS for (a) STF100 and (b) STF95.

Since we explicitly want to exclude cation segregation or Fe metal exsolution effects in this study, we evaluated the cation stoichiometry of the STF100 and STF95 film as a function of cell voltage. The cation stoichiometry is given in concentration p.f.u., meaning that Sr + Ti + Fe = 2 atoms per unit cell and shown as function of bias in Fig. 11. A slightly enhanced Sr concentration, almost nominal Ti stoichiometry and noticeable depletion in Fe are found, and the integral stoichiometry does not change with cell bias. Noteworthy, many studies in literature find much stronger Sr accumulation – especially after electrode testing in atmosphere.^47^ Interestingly, the difference between STF100 and STF95 is quite small, but still noticeable. A slight, reversible reduction of oxygen is observed in very reducing conditions (red symbols, right *y*-axis). However, the change of the absolute oxygen signal intensity is too weak to be reasonably quantified, as it lies in the range of experimental scatter. Also, the absolute O composition appears to be slightly too small. From the extrinsic doping, a stoichiometry of O_2.8_ would be expected for most of the *p*O_2_ range,^45^ but the deviation lies within the expected trustworthiness of XPS sensitivity factors from literature sources. Most importantly, the cation composition remains constant, indicating that no pronounced cation segregation occurred.

**Fig. 12:**
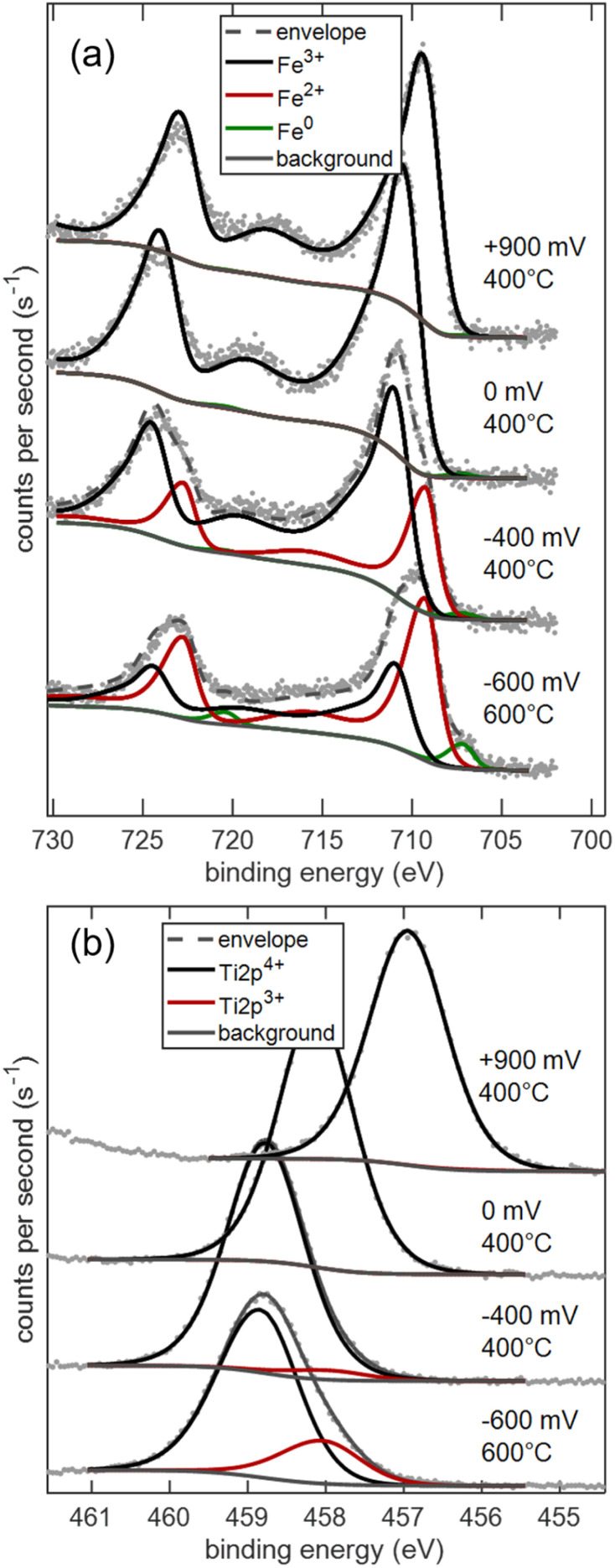
(a) Fe2p and (b) Ti2p_3/2_ peaks of the STF100 sample at different cell voltages and temperatures.

Much more pronounced changes with bias are observed when cation oxidation states are evaluated for Fe and Ti, plotted in Fig. 12. The spectra for STF100 are shown here, while (almost identical) spectra for STF95 are given the SI, Fig. S5. At +900 and 0 mV, the Fe2p spectra have the shape that is typical for Fe^3+^. When the cell potential is below 0 V, in accordance with literature ref. 48 and 49, the change in shape is indicative of the appearance of Fe^2+^. After the voltage step program at 400 °C, the sample was heated up to 600 °C, and reduced with a bias voltage down to −600 mV. Under these extremely reducing conditions, Fe metal (green component) appears, and also some Ti^3+^ becomes evident from the increasing asymmetry of the Ti2p_3/2_ peak. Analogously to GDC10, the shift of the Ti2p_3/2_ peak is caused by the dependence of the Fermi energy on the oxygen activity.^7^ A B.E. slope close to −1 eV V^−1^ was found and is shown in the SI. The Fe2p spectra were fitted as a linear combination of fingerprint patterns for Fe^3+^ (black), Fe^2+^ (red) and Fe^0^ (green), so that only four free fit parameters are used to describe the full peak shape. Measured XPS data and peak models are provided as CasaXPS Vamas files in the SI.

In the further discussion, we will only focus on the data acquired at 400 °C, where no Fe exsolution reactions occur.

When calculating the oxygen stoichiometry of STFO on the surface from transition metal oxidation states we can compare bulk and surface reducibility much more accurate than from evaluating the O1s intensity shown in Fig. 11. In a perovskite-type lattice that obeys electroneutrality, the O nonstoichiometry per formula unit (*δ*) is determined by the “missing” cation charges, relative to the nominal A^2+^ and B^4+^ cations, given by

11 3 − *δ* = 3 − 0.5[Ti^3+^] − 0.5[Fe^3+^] − [Fe^2+^] − 2[V_B_] − [V_Sr_] + 0.5[Fe_Sr_] + 0.5[h].

Therein, concentrations are given p.f.u. An absolute oxygen deficiency is hard to calculate for STF95, because bulk Sr deficiency may be compensated by Sr vacancies (additional acceptors) or Fe ions on the Sr site (donor defects). For the A : B site non-stoichiometric surface, this becomes even more complicated. Therefore, we just compare the relative change in stoichiometry *δ* − *δ*_0_. In accordance with literature^50,51^ set the reference cell voltage to +500 mV, where all ions have oxidation states Fe^3+^, Ti^4+^, Sr^2+^ and O^2−^, and we define our *δ*_0_. Consequently, we can use our XPS data to calculate the deviation from *δ*_0_ by

12 *δ* − *δ*_0_(XPS) = 0.5[Fe^2+^] + 0.5[Ti^3^] + 1.5[Fe^0^].

**Fig. 13:**
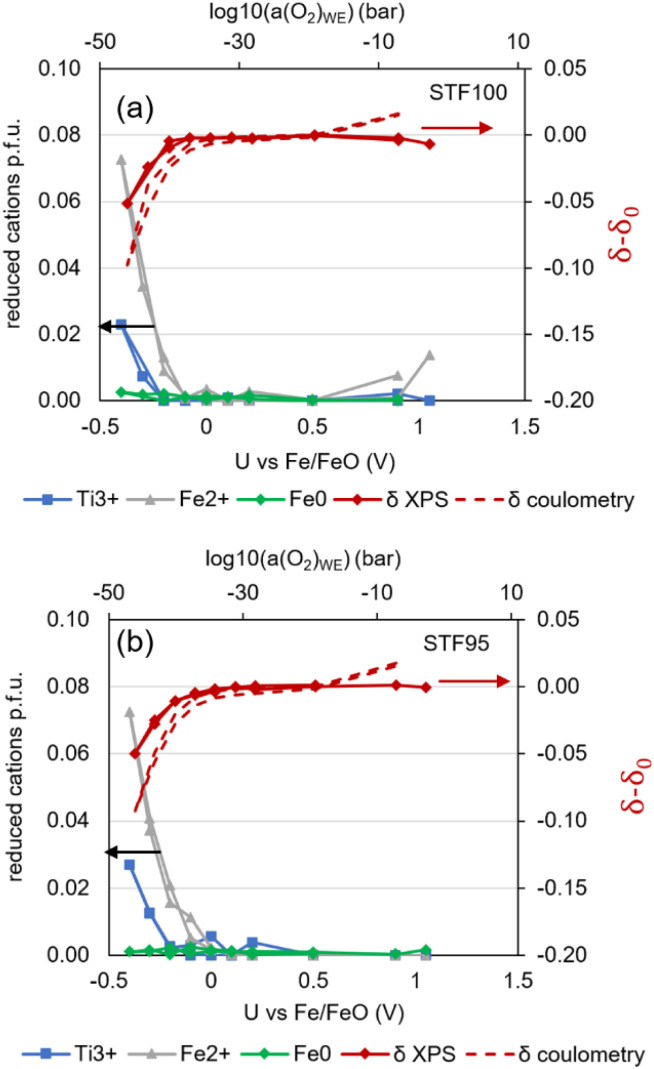
Concentrations of reduced cations (left *y*-axis, normalised as in Fig. 11 to Sr + Ti + Fe = 100% and change in oxygen stoichiometry (right *y*-axis) for (a) STF100 and (b) STF95.

Therein, concentrations were normalised to the actual surface stoichiometry found from the XPS quantification plotted in Fig. 11. The resulting plot of surface cation reduction and non-stoichiometry is shown in Fig. 13. It is clearly visible that a noticeable reduction of near-surface Fe starts at −100 mV, and some Ti^3+^ appears at −300 mV. Although a voltage of −400 mV *vs.* Fe/FeO corresponds to very reducing conditions, no Fe^0^ appears near the surface, presumably due to the slow cation diffusion at 400 °C. Comparison of the bulk nonstoichiometry from coulometry, using eqn (9), and the XPS-derived nonstoichiometry (eqn (12)) shows clearly that the surface is even slightly harder to reduce than the bulk of the material. A close look at the data shows that the fraction of Fe^2+^/Fe_tot_ is similar near the surface and in the bulk, but that only 60% of the nominal bulk Fe concentration is found near the surface. At +0.9 V, the coulometry data show a slight increase in the oxygen content. This is as expected, because the appearance of electron holes (sometimes interpreted as Fe^4+^ or O^−^) at high *p*O_2_ increases the oxygen content.^50,51^ These electronic defects, however, have no clear signature in XPS, so coulometry and XPS differ there.

Overall, we thus showed that STFO – and potentially many other perovskite-type oxides – is not easier to reduce near the surface. Rather, the near-surface Fe depletion makes the surface even harder to reduce. This has important implications for models of electrochemical reactivity or surface exsolution of transition metals. Also, A-site deficiency in STF95 has no significant effect on the reducibility or electrochemical stability of the material. Noteworthy, our data was taken at temperatures where cation migration is extremely sluggish. Likely, the temperature is too low for the cation diffusion that is required for the clustering of Fe metal atoms, so the structure remains metastable. Literature data acquired at higher temperature^7,16,45,52^ shows that STFO can exsolve catalytically active Fe nanoparticles and that A-site deficiency has a significant impact on the concentration of exsolutions.^46^

## 5. Conclusions

The surface chemistry of mixed ion and electron conductors redox–active transition metal ions depends strongly on the conditions in which the materials are used. Therefore, a large gap exists between UHV-based surface science and actual electrochemical or catalytic application of these materials. We could show that a properly designed solid oxide cell with MIEC thin film working electrode and an oxygen buffering Fe/FeO phase mixture in the counter electrode acts like an oxygen ion battery in vacuum. The Fe/FeO mixture is prepared *in situ* during an electrochemical counter electrode conditioning step and enables Electrochemical oXygen Activity ConTrol (EXACT) in the WE. The range of continuously controllable oxygen activity reaches from the electrolyte reduction limit (*ca.* 10^−45^ bar at 600 °C), and is limited by oxygen O_2_ release, which becomes noticeable above *ca.* 10^−7^ bar. At lower temperature, *ca.* 300 °C, oxygen release is slowed down and even enables oxygen activities corresponding to more than 1 bar. Also, the temperature window is very broad, ranging from *ca.* 300 °C (limited by the conductivity of YSZ) up to >1000 °C, typically limited by the used heater. In addition to oxygen activity control, bulk oxygen stoichiometry changes of the WE can be precisely monitored by simultaneous coulometric titration measurements.

Proof-of-concept XPS measurements were performed on cells containing Ce_0.9_Gd_0.1_O_1.95−*δ*_ (GDC), SrTi_0.6_Fe_0.4_O_3−*δ*_ (STF100) and Sr_0.95_Ti_0.6_Fe_0.4_O_3−*δ*_ (STF95) thin film working electrodes. We could show that electrochemical oxygen activity control even enables measurements beyond the limits of classical gas mixtures, and go to much more reducing conditions. On GDC, we found enhanced surface reducibility, in excellent quantitative agreement with previous ambient pressure XPS measurements.^11^ This proves that the broken symmetry at the surface is responsible for easier reduction, rather than surface OH groups. Additional features in the electrochemical oxygen nonstoichiometry data indicate the appearance of vacancy-ordered phases in very reducing conditions that would be unachievable by gas mixtures.

For the perovskite-type materials STF100 and STF95, in contrast to GDC, the surface is harder to reduce. This is likely linked to a surface depletion of reducible Fe cations that was found for the freshly deposited films. Furthermore, surprisingly high phase stability was observed in highly reducing conditions that even cause partial reduction of Ti^4+^ to Ti^3+^. This stability is probably due to the low testing temperature of 400 °C at which cations are not expected to be mobile.^53^

As an outlook, this paper only contains XPS measurements, but the EXACT method for oxygen activity control is compatible with any other UHV-based analytic technique, and we are confident that the combination of more analytic UHV methods will enable new insights into catalytically active surfaces close to operation conditions. For example, with Low Energy Ion Scattering (LEIS), changes in the surface oxygen vacancy concentration or potential voltage dependent cation segregation may be investigated. With Secondary Ion Mass Spectroscopy (SIMS) one may investigate how oxidation and reduction of the oxide influence the matrix effects in elemental quantification. Also, SEM investigations of morphological changes (*e.g.* transition metal exsolution from Perovskite-type electrode materials, or chemical expansion) would be highly interesting to observe, just to name a few of the possibilities opened with this technique.

## Conflicts of interest

There are no conflicts to declare.

## Supplementary Material

TA-013-D5TA02648B-s001

TA-013-D5TA02648B-s002

## Data Availability

Supplementary information is available. See DOI: https://doi.org/10.1039/d5ta02648b. It contains a .pdf file with additional electrochemical results and XPS spectra. Furthermore, all XPS data collected and presented for this study are available in Vamas format (readable *e.g.* by CasaXPS) in the supplementary information (.zip file). The structure of the data sets is explained in the supplementary pdf file.
