# Supplementary material for: UHV-based analytics with electrochemical oxygen activity control
Source: J Mater Chem A Mater. 2025 Jul 31;13(35):29147–60. doi: 10.1039/d5ta02648b (PMC12326307; doi:10.1039/d5ta02648b)
Supplement: TA-013-D5TA02648B-s001 [file TA-013-D5TA02648B-s001.pdf]

Supplementary Information (SI) for Journal of Materials Chemistry A.
This journal is © The Royal Society of Chemistry 2025

Supporting information for

UHV-based analytics with electrochemical oxygen activity control

Andreas Nenning, Stanislaus Breitwieser, Christian Melcher, Jürgen Fleig

Institute of Chemical Technologies and Analytics, Research Group for Electrochemical Energy Conversion, TU, Wien, Austria

Correspondence: andreas.nenning@tuwien.ac.at;

1. EIS characterization for temperature monitoring

The sample temperature was determined from the temperature dependent conductivity of the used YSZ substrates[1]. Impedance spectra are plotted below in Figure S1. Before and after conditioning the high frequency axes intercept of 260 Ω is unchanged, and corresponds to a temperature of 510°C. Due to the lack of an atmosphere, the electrode feature resembles a capacitor. After CE conditioning, the oxygen activity in the cell is lowered, and thus the chemical capacitance increases, leading to a substantially smaller capacitive impedance at 0.1 Hz.

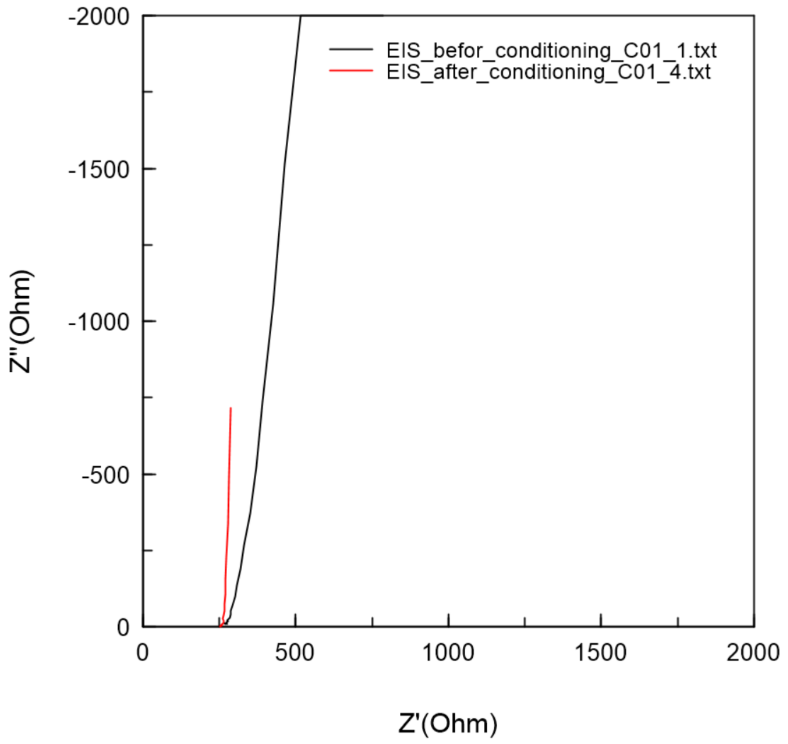

Figure S1. Electrochemical impedance spectra of GDC10 at 0V cell voltage, before (black) and after (red) conditioning.

2. Peak shift due to overpotential variation

As exemplified in literature, the Fermi energy of STFO and GDC varies with the oxygen activity, and thus the cell overpotential. Since the measured "binding energy" in XPS is actually the core level-Fermi level difference, a systematic dependence of measured binding energy and cell voltage is expected (although the WE is grounded). In an idealised point defect model that assumes that oxygen vacancies are more numerous than reduced ions, this results in the relation[2]

$$BE = BE^0 - e * U_{cell},$$

where BE^0 is the binding energy at 0V cell voltage.

Shown in Figure S2, this slope is almost met. This plot also shows that for STFO, the Ti2p3/2 binding energy is a good anchor for the oxygen chemical potential, as the data for STF95 and STF100 for 400 and 600°C lie on the same curves. For estimating the maximum achievable oxygen activity with the EXACT method, the cell temperature was decreased to 280°C, and an extremely high potential of +9V was applied to the cell. In these conditions, most of the cell voltage is the electrolyte overpotential drop, and oxygen is released with a rate of ~100µA/cm^2. The Ti2p3/2 binding energy measured at these conditions (456.8 eV) can serve as a reference for the degree of STFO oxidation, and intercepts linear regression line at a voltage of 1.28 V, or an oxygen activity of 100 bars. Thus, at sufficiently low temperature it is even possible to study the material in a highly oxidized state in which O_2 is slowly leaking into the chamber. The CE oxygen buffering capacity allows this for roughly 4 hours.

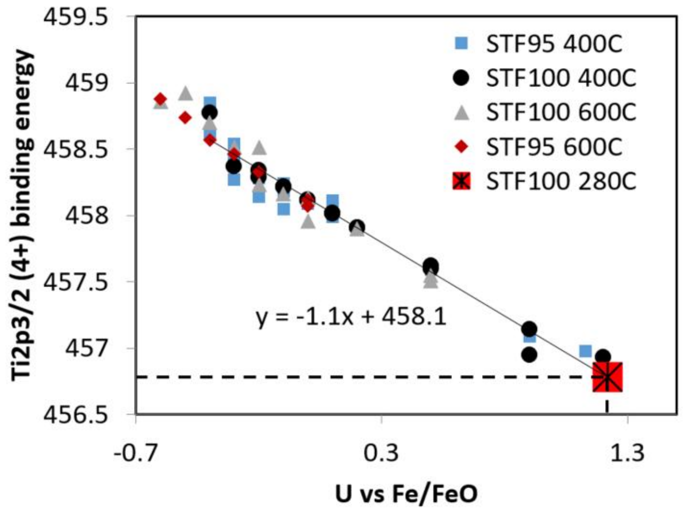

Figure S2. Binding energy of the Ti2p 3/2 peak as function of cell voltage for STF95 an STF100.

3. O1s XPS spectra of GDC10

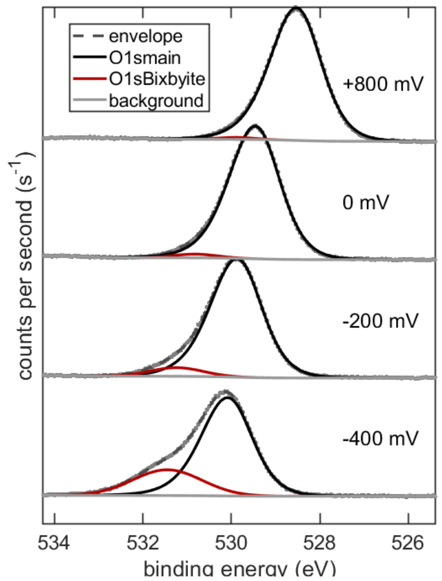

Figure S2. O1s spectra of GDC10 at 510°C and various potentials vs Fe/FeO. An additional species (red) appears under very reducing conditions.

4. Electrical characterisation of STF100

Analogously to the data shown in Figure 9 in the original manuscript, coulometry was used to calculate the bulk oxygen stoichiometry change as function of voltage. Due to a typo in the automated measurement sequence, the holding time at -0.3 V (decreasing voltage direction) was 30 seconds, not 30 minutes.

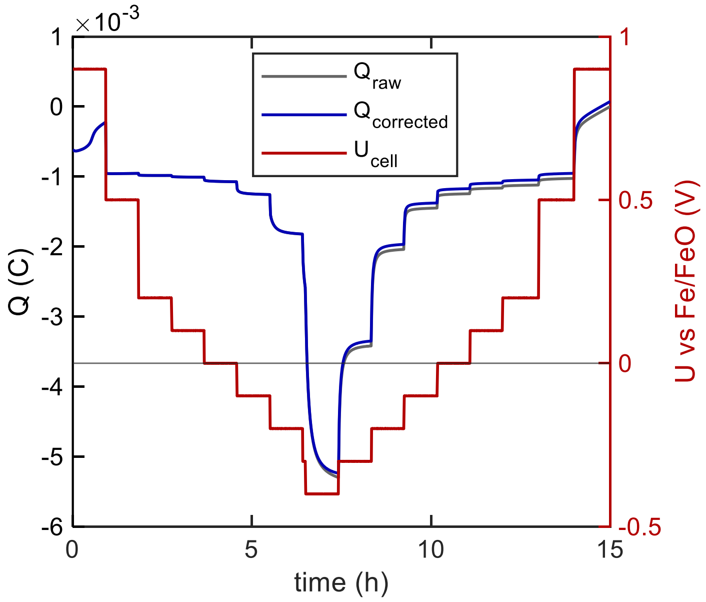

Figure S3. Electrical testing of the STF100 sample at 400°C.

5. Additional XPS spectra of STF95 and STF100

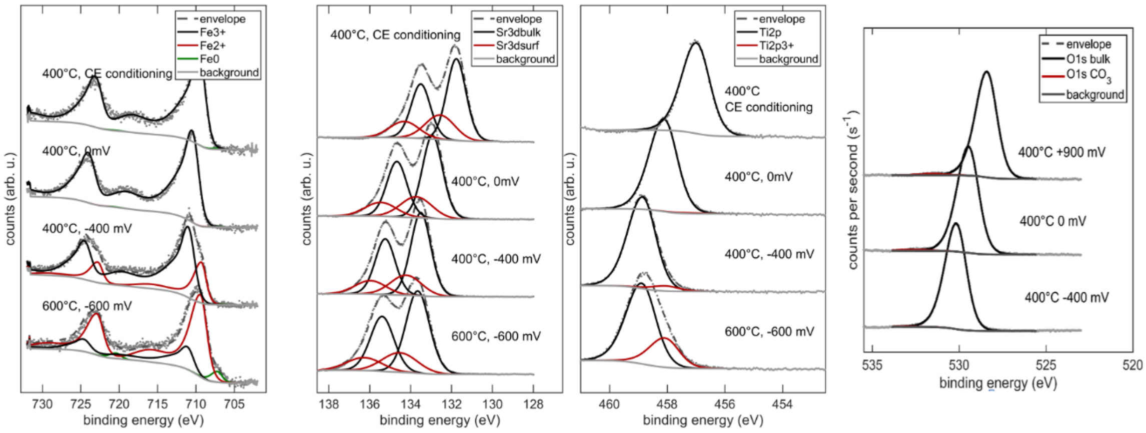

Figure S4. Fe2p, Sr3d, Ti2p3/2 and O1s XPS spectra of STF95 as function of cell voltage at 400°C and 600°C.

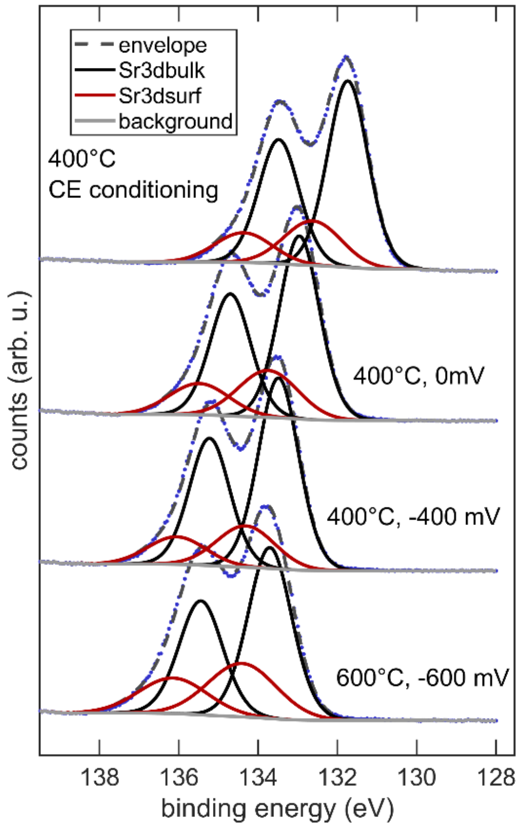

Figure S5. Sr3d spectra of STF100 as function of cell voltage at 400°C.

6. Structure of the supplementary Vamas files

Raw data and peak models used in this study are provided as VAMAS files (readable with CASAXPS, and other software. One file is provided per sample (GDC10, STF95 and STF100).

The “Sample Identifier” row contains the applied cell potential, or the information “CE conditioning” (data recorded during CE conditioning, and hence most oxidizing conditions achievable).

Researchers are encouraged to use the provided peak models in fitting their own data.

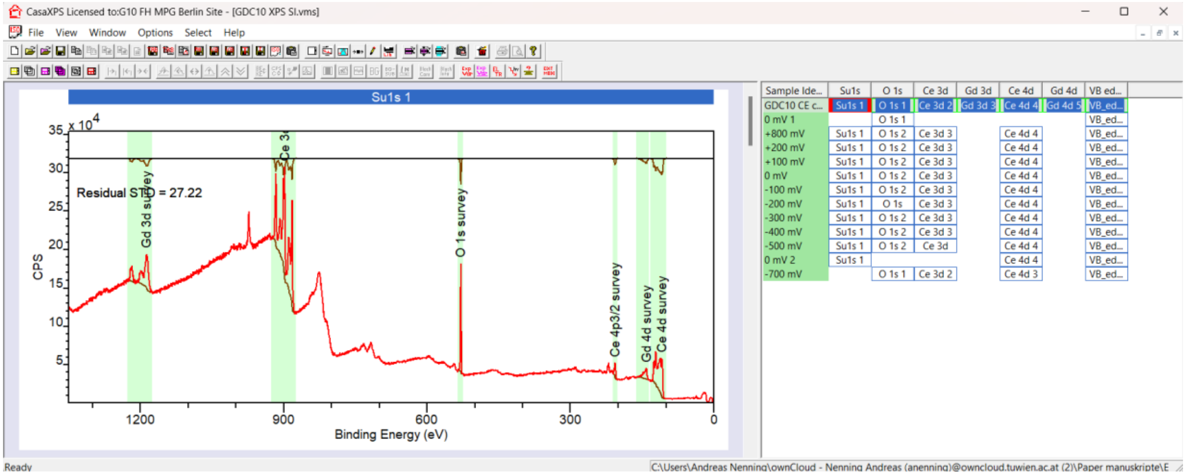

Figure S6. Screenshot of the Vamas file for GDC10.

7. Calculation of sensitivity factors

Unfortunately, the numerically derived Scofield[3] atomic sensitivity factors from year 1976 that are embedded in CASAxps are very inaccurate for chemical quantification.

Sensitivity factors published by Brundle and Christ[4], https://xpslibrary.com/%CF%83-sf-asf-and-rsf/ that use reference compounds (Fe2O3, TiO2, CeO2, SrCO3) provide much more consistent results. However, the Versaprobe instrument has a 45° source-analyser angle, whereas the reference cross sections were collected at the magic angle of 54.7°. The asymmetry factor of the photoemission process thus influences the detected photoelectron intensity.

Asymmetry values β were taken from the web services of the Elettra Trieste synchrotron (https://vuo.elettra.eu/services/elements/WebElements.html), and cross sections were corrected by multiplying them with the 2nd order legendre polynomial that describes the angular distribution of photoelectrons[5]

$$SF_{45} = SF_{Christ} * \left(1 - 0.25\beta\left(3cos(\Theta)^2 - 1\right)\right)$$

Therein, SF45 is the used sensitivity factor of the Versaprope, SF_{christ} the empirical sensitivity factor from an instrument with magic angle geometry, and θ the source-analyser angle (45°).

The Ce3d SF is a special case, because a Tougaard background was used in the peak model, whereas the empirical SFs, were determined with Shirley background, which results in ~30 % more peak area for the Tougaard background. For comparability, the Ce3d SF was thus multiplied by 1.3.

8. CV sweeps of a STFO thin film electrode that contains FeO_x impurities (confirmation of the CE stability)

In order to proof that the oxygen activity in the CE remains at the Fe/FeO equilibrium, multiple CV sweeps and Ti 2p XPS spectra (at 0 mV cell voltage) of a model cell with $SrTi_{0.3}Fe_{0.7}O_{3-\delta}$ working electrode were acquired over a duration of 10 hours. The CV curves show a capacitive loop, and small distinct redox peaks corresponding to the oxidation and reduction of FeO_x impurities that were present as an artifact of imperfect PLD deposition. Clearly visible, the position of these peaks remains stable over the duration of the experiments. Also the B.E. of the Ti2p peak is constant, indicating that the oxygen activity does not change (in contrast to the shift observed in Fig. S2).

The oxidation and reduction peaks are shifted by 50 mV. This difference has been identified in literature before, and attributed to a nucleation overpotential for the phase transition [6]. Consequently, this result supports the stability of the CE potential.

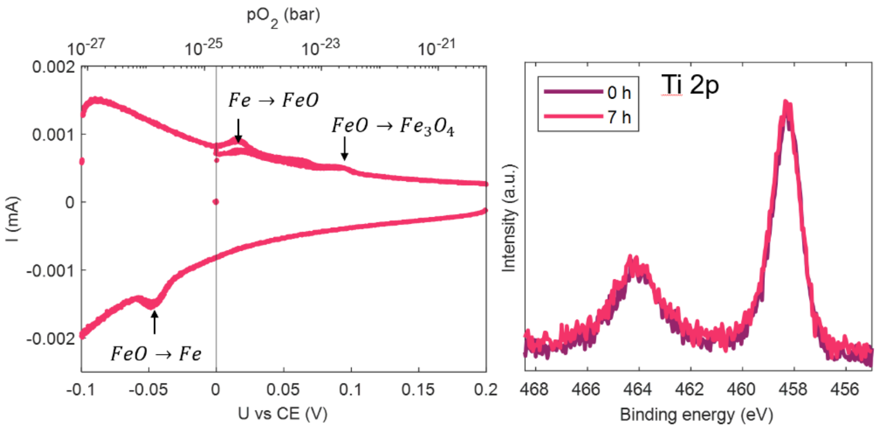

Figure S8. CV sweep of a STFO thin film containing small FeO_x impurities, acquired at 600°C with a sweep rate of 1 mV/s. The redox processes FeO_x impurities and their corresponding potentials are annotated in the plot.

9. References

1. Ahamer, C.; Opitz, A.K.; Rupp, G.M.; Fleig, J. Revisiting the Temperature Dependent Ionic Conductivity of Yttria Stabilized Zirconia (YSZ). *J. Electrochem. Soc.* **2017**, *164*, F790–F803, doi:10.1149/2.0641707jes.
2. Nenning, A.; Opitz, A.K.; Rameshan, C.; Rameshan, R.; Blume, R.; Hävecker, M.; Knop-Gericke, A.; Rupprechter, G.; Klötzer, B.; Fleig, J. Ambient Pressure XPS Study of Mixed Conducting Perovskite-Type SOFC Cathode and Anode Materials under Well-Defined Electrochemical Polarization. *J. Phys. Chem. C* **2016**, *120*, 1461–1471, doi:10.1021/acs.jpcc.5b08596.
3. Scofield, J.H. Hartree-Slater Subshell Photoionization Cross-Sections at 1254 and 1487 eV. *J. Electron Spectrosc. Relat. Phenom.* **1976**, *8*, 129–137, doi:10.1016/0368-2048(76)80015-1.
4. Brundle, C.R.; Crist, B.V. X-Ray Photoelectron Spectroscopy: A Perspective on Quantitation Accuracy for Composition Analysis of Homogeneous Materials. *J. Vac. Sci. Technol. A* **2020**, *38*, 041001, doi:10.1116/1.5143897.

5. Jablonski, A.; Zemek, J. Overlayer Thickness Determination by XPS Using the Multiline Approach. *Surf. Interface Anal.* **2009**, *41*, 193–204, doi:10.1002/sia.3005.
6. Summerer, H.; Nenning, A.; Rameshan, C.; Opitz, A.K. Exsolved Catalyst Particles as a Plaything of Atmosphere and Electrochemistry. *EES Catal.* **2023**, *1*, 274–289, doi:10.1039/D2EY00036A.
